# The variome concept: focus on CNVariome

**DOI:** 10.1186/s13039-019-0467-8

**Published:** 2019-12-19

**Authors:** Ivan Y. Iourov, Svetlana G. Vorsanova, Yuri B. Yurov

**Affiliations:** 1 Yurov’s Laboratory of Molecular Genetics and Cytogenomics of the Brain, Mental Health Research Center, 117152 Moscow, Russia; 2 Veltischev Research and Clinical Institute for Pediatrics of the Pirogov Russian National Research Medical University, Ministry of Health of Russian Federation, 125412 Moscow, Russia

**Keywords:** Copy number variations, Genome variations, Pathways, Somatic mosaicism, Variome

## Abstract

**Background:**

Variome may be used for designating complex system of interplay between genomic variations specific for an individual or a disease. Despite the recognized complexity of genomic basis for phenotypic traits and diseases, studies of genetic causes of a disease are usually dedicated to the identification of single causative genomic changes (mutations). When such an artificially simplified model is employed, genomic basis of phenotypic outcomes remains elusive in the overwhelming majority of human diseases. Moreover, it is repeatedly demonstrated that multiple genomic changes within an individual genome are likely to underlie the phenome. Probably the best example of cumulative effect of variome on the phenotype is CNV (copy number variation) burden. Accordingly, we have proposed a variome concept based on CNV studies providing the evidence for the existence of a CNVariome (the set of CNV affecting an individual genome), a target for genomic analyses useful for unraveling genetic mechanisms of diseases and phenotypic traits.

**Conclusion:**

Variome (CNVariome) concept suggests that a genomic milieu is determined by the whole set of genomic variations (CNV) within an individual genome. The genomic milieu is likely to result from interplay between these variations. Furthermore, such kind of variome may be either individual or disease-specific. Additionally, such variome may be pathway-specific. The latter is able to affect molecular/cellular pathways of genome stability maintenance leading to occurrence of genomic/chromosome instability and/or somatic mosaicism resulting in somatic variome. This variome type seems to be important for unraveling disease mechanisms, as well. Finally, it appears that bioinformatic analysis of both individual and somatic variomes in the context of diseases- and pathway-specific variomes is the most promising way to determine genomic basis of the phenome and to unravel disease mechanisms for the management and treatment of currently incurable diseases.

*— You should always count everything, because everything counts.*

*Raymond M. Smullyan*

*(Alice in Puzzle-Land —*

*A Carrollian Tale for Children under Eighty)*

Variome is a term, which is generally used in the context of The Human Variome Project dedicated to collecting and integrating genomic data for the identification of disease-causing genome variations [1]. On the other hand, variome may be defined as a complete (near-complete) set of genomic variations in an individual (individual variome or V_i_). Alternatively, a set of genomic variations associated with specific phenotypic traits or disease/condition may be described as a trait-specific or disease-specific variome (V_ds_). Actually, almost all relevant studies of disease-causing genetic changes in the whole genomic context might be designated as “variome analysis”, inasmuch as these studies are targeted at uncovering the whole set of genomic variations associated with a specific phenotype. For instance, genome-wide association studies (GWAS) is a picturesque, albeit not always successful, example of establishing V_ds_ or the variome specific for a phenotypic trait/condition at the sequence level. Roughly, one can subdivide a general variome (V_i_ or V_ds_) into two large distinct “variomic subtypes”: sequence variome (all the genomic variations detectable at the sequence level) and variome encompassing all the copy number variations (CNVs) or “CNVariome” adding balanced/imbalanced chromosome rearrangements and chromosomal heteromorphisms. Here, we focus on CNVariome to present the variome concept, which is able to become a useful system for high-throughput analysis of the functional consequences of individual and disease-specific genomic variability.

CNVs have long been observed to contribute to genetic diversity in health and disease [2–5]. However, despite significant advances in CNV biology, difficulties in describing phenotypic outcome of both rare and common CNVs require an extensive bioinformatic analysis for identifying the pathogenic value (if any) of a genomic change from an individual CNVariome [6, 7]. An average individual genome exhibits myriads of sequence variants and hundreds of CNVs; bioinformatic analysis seems to be indispensable for evaluating each variant for defining the contribution to the phenotype [8, 9]. To succeed in gene/CNV prioritization or systems biology (OMICS/pathway-based) analysis, a big data analysis of genomic variations performed at genomic, epigenetic, proteomic and metabolome levels is applied [7, 10–12]. Moreover, recent studies have shown that CNVs are able to interact between each other within V_i_ in a complex and multilateral fashion [13]. Therefore, systems biology analyses of genomic variations suggest that causative variants are not isolated. Indeed, since genome variations are able to either increase or decrease the effect of each other, V_i_ may be considered as a kind of “genomic milieu” for disease-causing (trait-associated) variations.

The assumption that CNVariome is a genomic milieu modulating functional effects of CNVs may be further supported by the observation on the so-called “CNV burden” (a fraction of CNVs in an individual genome with appreciable functional consequences) [14–16], which perfectly fits the variome concept or, more exactly, the CNVariome concept. Furthermore, experimental testing of two- and multiple-hit hypothesis has formed a firm basis for the CNVariome concept. According to the hypothesis, CNVs are able to increase or decrease gene dosage creating a genomic background or milieu (the first hit). This milieu is highly sensible to subsequent “second hit” or “multiple hits” (i.e. sporadic mutations, somatic mosaicism or chromosome/genome instability), which produces specific phenotypes [17–20]. Therefore, to define the genomic milieu created by CNVariome, it is to address all detectable CNVs in an individual genome. These data would be important for the definition of V_ds_, as well.

The phenotypic effect of genomic variations is achieved through the impact on specific molecular and cellular pathways. Although the spectrum of functional consequences of genomic variations seems to be extremely broad, it is generally acknowledged that an effect (even if it is low) on molecular pathways/processes does exist [12, 21–23]. Additionally, common variants are able to possess functional effects at the protein level [22]. Therefore, to address genomic variations’ impact on molecular pathways, one has to take into account the whole set of variants detected in an individual genome or, in other words, one has to analyze the variome. As to CNVs (CNVariome), our previous studies have shown that both common CNVs with a slight functional effect on a pathway (e.g. CNVs affecting genes involved in the cell cycle pathway) are able to cause chromosome instability detectable by cytogenetic analysis [20, 24, 25]. Similar results have been reported in studies of variome at the sequence level in health and disease highlighting new genomic mechanisms for interindividual diversity achieved through the functional variation in molecular pathways [26, 27]. More importantly, it is suggested that such genomic variations contribute to intercellular functional diversity, as well [28–30]. Genome variability/instability in cancer is probably the best example of how multiple chromosome abnormalities, CNVs and/or gene mutations may lead to dramatic functional changes of molecular and cellular pathways [31–33]. Actually, single-cell systems biology analyses have shown that such pathway changes mediated by genomic variations do occur [34, 35]. Therefore, it is useful to introduce another “variomic subtype”, i.e. pathway-specific variome(s) (V_ps_) covering the set of genomic variations affecting specific pathway. Thus, V_i_ or V_ds_ appears to be composed of a number of V_ps_. Taking into account how CNVs functionally affect molecular and cellular pathways mediating the phenotypic outcome [12–14, 16–19, 35–37], one can suggest that appreciable effects of V_ps_ are likely to be the result of a number of variants that affects the pathway. In other words, the effect results from a kind of saturation in genomic variations or in CNVs. Figure 1 schematically depicts the variome (CNVariome) concept in the light of functional effects of genomic variations on molecular and cellular pathways.

**Fig. 1:**
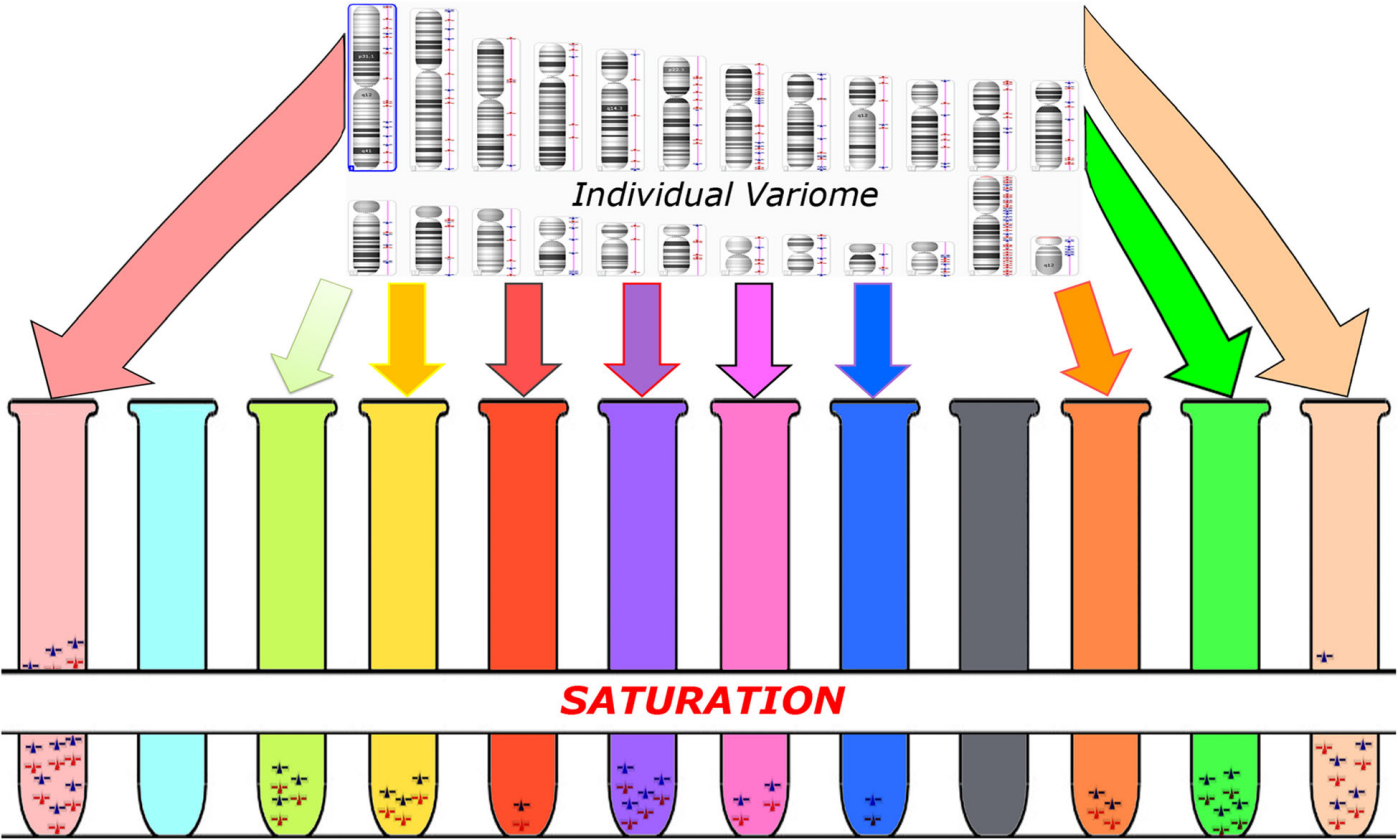
Schematic representation of the variome (CNVariome) concept; a set of genomic variations (e.g. CNV) may contribute to functional variability of molecular/cellular pathways or create several V_ps_ symbolically depicted as colored laboratory tubes; when the number of genomic variations achieves a critical level (i.e. saturation in genomic variations), an alteration to the pathway occurs (e.g. left-most and right-most tubes). A screenshot of CNV analysis by Chromosome Analysis Suite (ChAS) 3.10.15© 2015 Affymetrix Inc. was used to depict individual variome (CNVariome)

Molecular pathways are the intermediate link between the genome and the phenome [13, 23]. Moreover, the pathways are promising targets for molecular-oriented therapy in diseases associated with genomic pathology (i.e. chromosomal abnormalities and CNVs) [29, 38–40]. Therefore, the identification of V_ps_ or decomposition of V_i_/V_ds_ into a set of V_ps_ would be useful for unraveling disease mechanisms to develop effective management and treatment of currently incurable genetic diseases.

An important aspect of genomic variability is referred to somatic genome variations or somatic mosaicism. Indeed, this phenomenon reflect an important, albeit commonly overlooked, ability of genome (cellular genomes) to vary through ontogeny [37, 41]. Somatic mutations cause cancer [31–34, 42]. Somatic mosaicism has been consistently associated with interindividual/intercellular genomic heterogeneity in health and disease [43–45]. More precisely, neurodevelopmental diseases have been repeatedly associated with chromosomal mosaicism and mosaic CNVs/gene mutations [37, 46–49]. Chromosomal mosaicism and instability has been demonstrated to mediate neurodegeneration [48–57]. Further studies have indicated that chromosome instability is an important element of a pathogenic cascade of neurodegenerative diseases [52, 54–57]. Therefore, to address genomic (genetic) mechanisms for human diseases in their complexity, it is unavoidable to consider somatic genome variations. In the light of the variome concept, we introduce the somatic variome (V_s_), which may be defined as a set of somatic genome variations (somatic mosaicism + genomic and chromosomal instability) detected in an individual. Somatic mosaicism and genomic/chromosomal instability are likely to occur due to altered molecular/cellular pathways controlling genome stability maintenance, cell cycle and programmed cell death [18, 24, 31, 42, 45, 57]. We hypothesize that a set of V_ps_ of these pathways is able to produce a kind of susceptibility of cellular genomes to genomic/chromosomal instability and/or somatic mosaicism. Therefore, systems biology analyses of V_i_ and molecular cytogenetic survey of chromosome/genome instability may identify the link between non-mosaic (germline inherited/sporadic) and somatic genome variations. Figure 2 shows the interplay between V_i_ and V_s_ according to the concept. The result of addressing the interplay between V_i_ and V_s_ might be a “constitutional variome”, which might be basic genomic background of all the somatic cells evolved/developed from single zygote encompassing acquired somatic mutations in different tissues produced by genetic-environmental interactions and ontogenetic genome variations. The “constitutional variome” might represent a complex system of interactions between the whole set of individual and intercellular genome variations uniquely describing the intrinsic genomic milieu of an individual. The genomic milieu should reflect the whole burden of genomic variations including individual combinations of inherited variants, which are able to modulate the phenotype (i.e. reduced penetrance or increased severity of disease manifestations and phenotypic traits) or, in other words, to form an “inheritance pattern” applicable not only to monogenic diseases, but also to multifactorial disorders in a threshold manner as shown in Fig. 1.

**Fig. 2:**
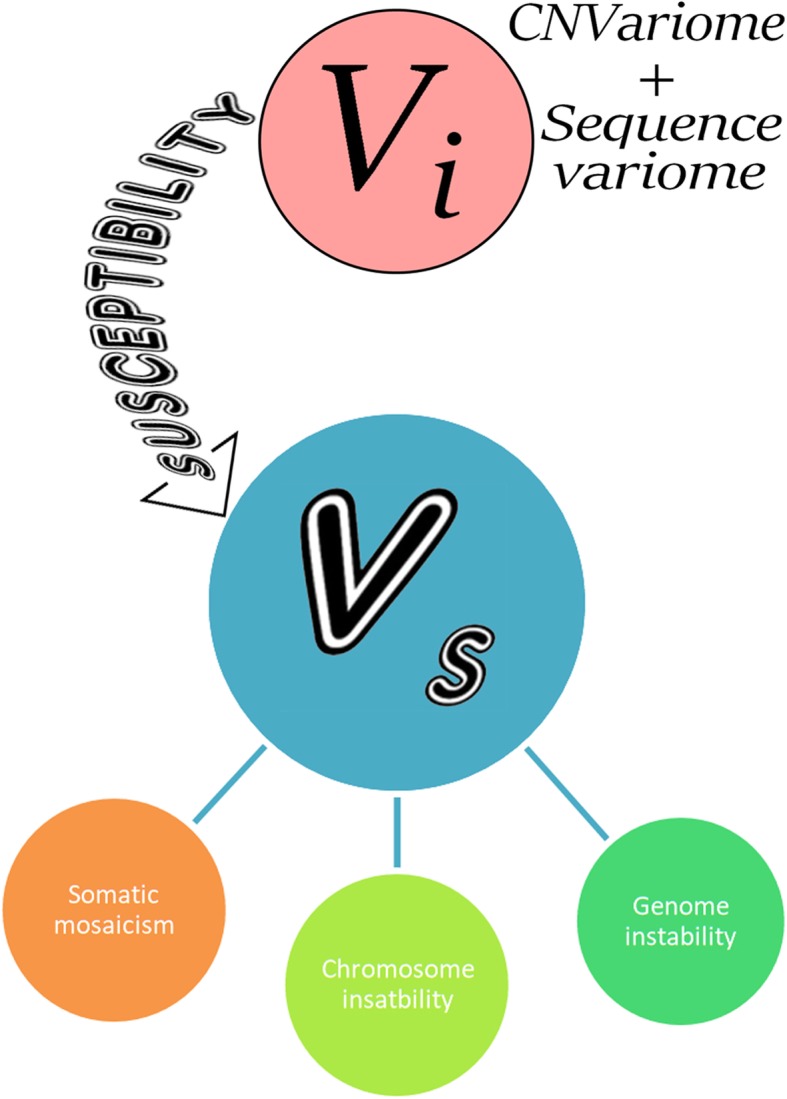
Interplay between V_i_ and V_s_; CNVariome and sequence variome forming V_i_ may alter pathways critical for maintaining genome stability, cell cycle, and/or programmed cell death; the result of such alterations is likely to be a susceptibility for specific V_s_, which encompass somatic mosacism as well as chromosome and genome instability

The variome concept proposes that functional variability of molecular/cellular pathways determining the phenome is the result of the effect of all the variants of an individual genome (variome). The effects can be cumulative, interchangeable, mutually exclusive or neutral. However, one should bear in mind that the whole V_i_/V_ds_ should be addressed to determine the intrinsic effect. In the genomic context, V_i_ and V_ds_ are composed of two variome subtypes: sequence variome and CNVariome. In the functional genomic context, V_i_ /V_ds_ may be decomposed into a set of V_ps_. In the (molecular) cytogenetic context, the CNVariome concept suggests that the individual uniqueness at molecular and cellular levels may result from an individual set of CNVs (in addition to sequence variome). If V_i_/V_ds_ is enriched in V_ps_ of genome stability maintenance, cell cycle, and programmed cell death, a susceptibility to genomic/chromosomal instability and/or somatic mosaicism may occur. Accordingly, V_s_ should be addressed in addition to V_i_/V_ds_ for comprehensive evaluation of the whole spectrum of genomic changes contributing to the phenome. Since the famous quote “Ontogeny recapitulates phylogeny” from Ernst Haeckel’s classic works remains more-or-less actual [58–60], variome concept encompassing V_i_/V_ds_, V_ps_ and V_s_ seems to applicable in the evolutionary context, as well, allowing us to finalize description of the concept by another famous quotation “Nothing in biology makes sense except in the light of evolution” from Theodosius Dobzhansky [61]. To this end, we believe that the concept is able to provide a valuable system for determining the genomic basis of the phenome and for unravel disease mechanisms to succeed in the management and treatment of currently incurable genetic diseases.

## Data Availability

Not applicable.

## Abbreviations

CNVs: Copy number variations
GWAS: Genome-wide association studies
V_ds_: Disease-specific variome
V_i_: Individual variome
V_ps_: Pathway-specific variome
V_s_: Somatic variome
